# Supplementary figures and images for: Anti-inflammatory effects of elexacaftor/tezacaftor/ivacaftor in adults with cystic fibrosis heterozygous for F508del
Source: PLoS One. 2024 May 31;19(5):e0304555. doi: 10.1371/journal.pone.0304555 (PMC11142445; doi:10.1371/journal.pone.0304555)

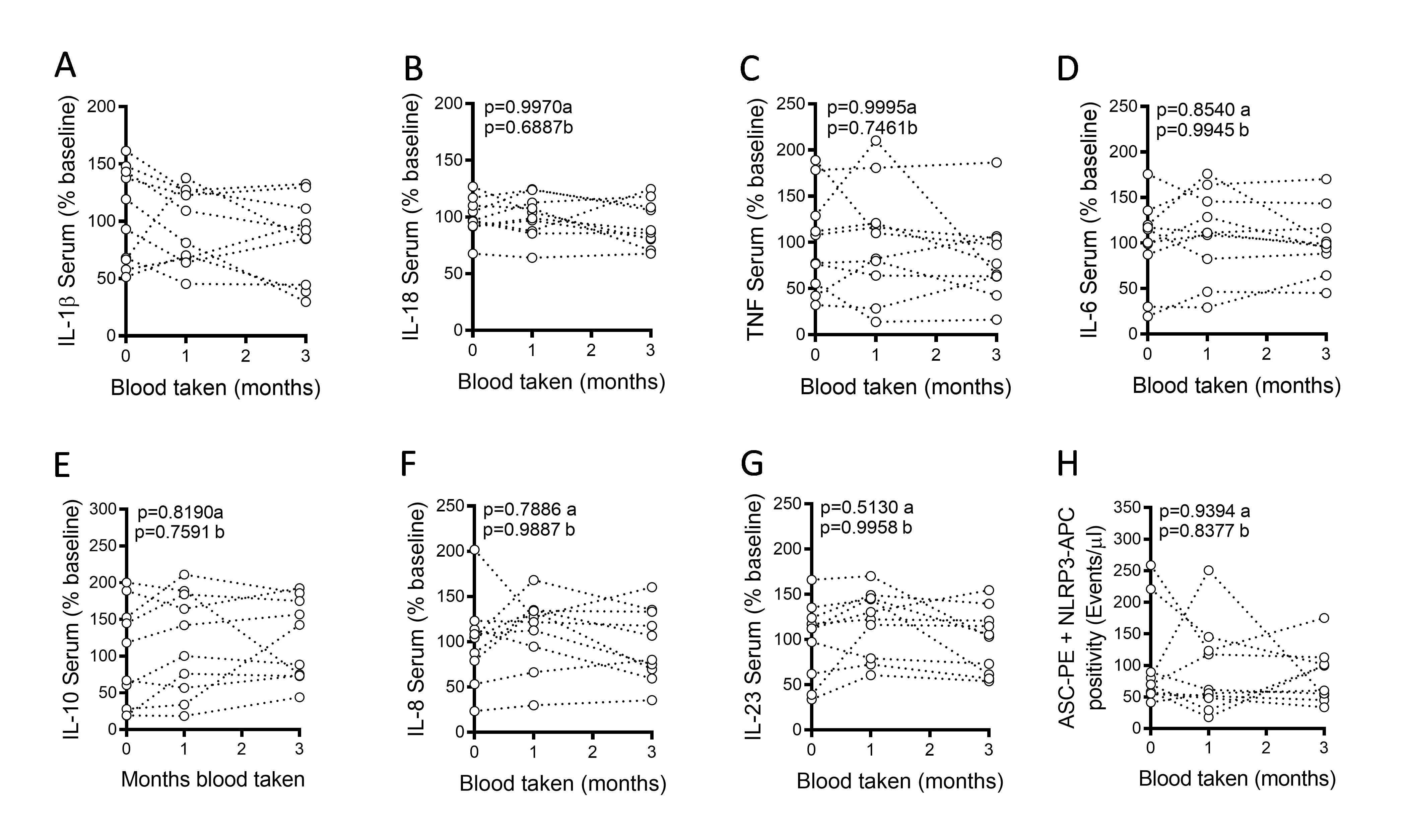

Supplement: S1 Fig — Sera were collected at zero-month, one month and three month from HC (n = 10). LegendPlex assays were used to detect levels of A, IL-18; B, IL-1β; C, TNF; D, IL-6; E, IL-10; F, IL-8; G, IL-23 in serum. H, Flow cytometry was used to detect NLRP3 positive ASC-specks. A one-way ANOVA statistical test with Tukey’s multiple comparison was performed. P value for baseline to one month (a) and baseline to three months (b) shown on each graph. (TIF) [file pone.0304555.s001.tif]

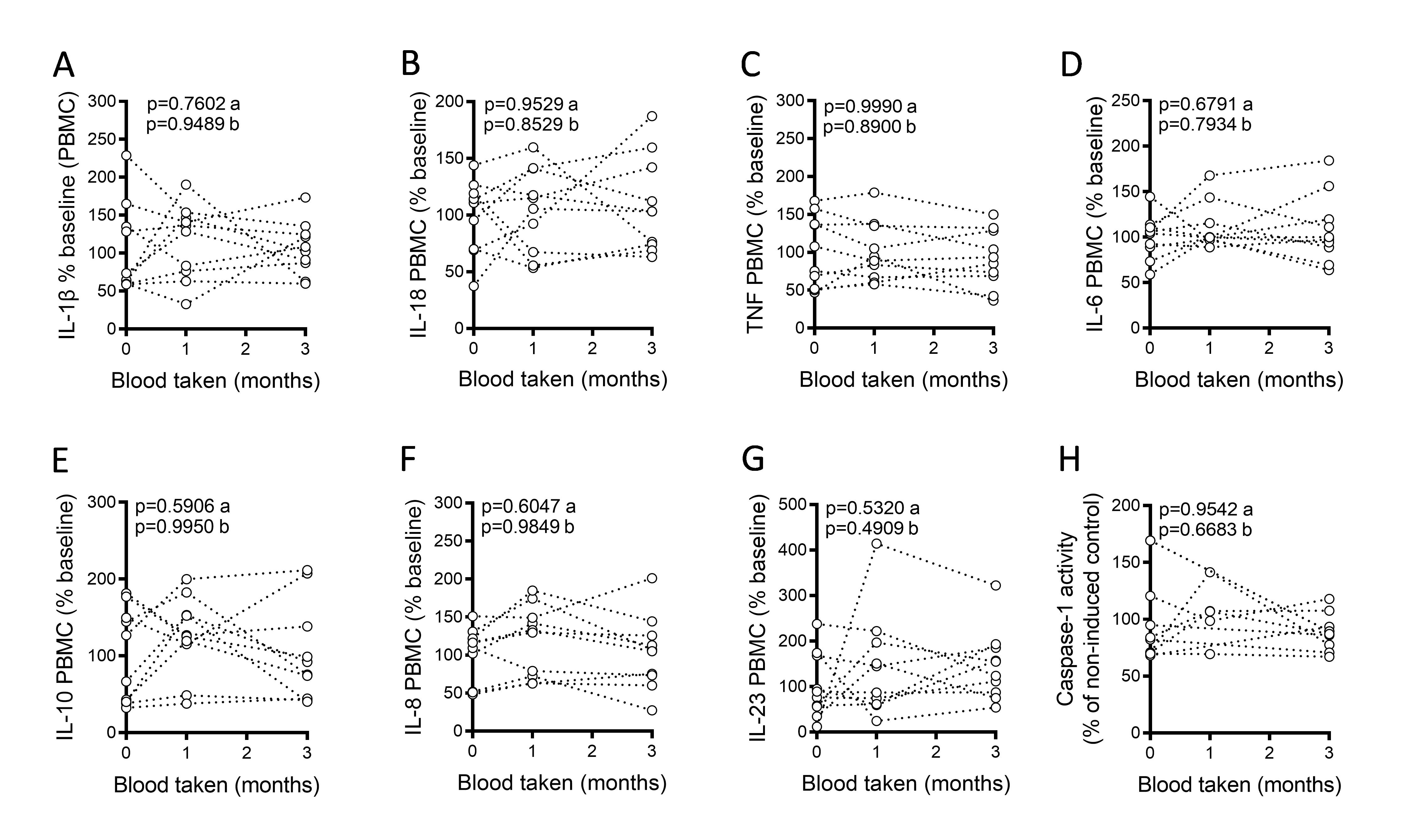

Supplement: S2 Fig — Sera were collected at zero-month, one month and three month from HC volunteers (n = 10). Following isolation, PBMCs were immediately stimulated with LPS (10ng/mL, 4hr), and ATP (5mM) for the final 30 min. LegendPlex assays were used to detect levels of A, IL-1β; B, IL-18; C, TNF; D, IL-6; E, IL-10; F, IL-8; G, IL-23 secretion from PBMCs. A Caspase-1 activity was detected in stimulated PBMCs at each time point. A two-way ANOVA statistical test with Tukey’s multiple comparison was performed. P value for baseline to one month (a) and baseline to three months (b) shown on each graph. (TIF) [file pone.0304555.s002.tif]

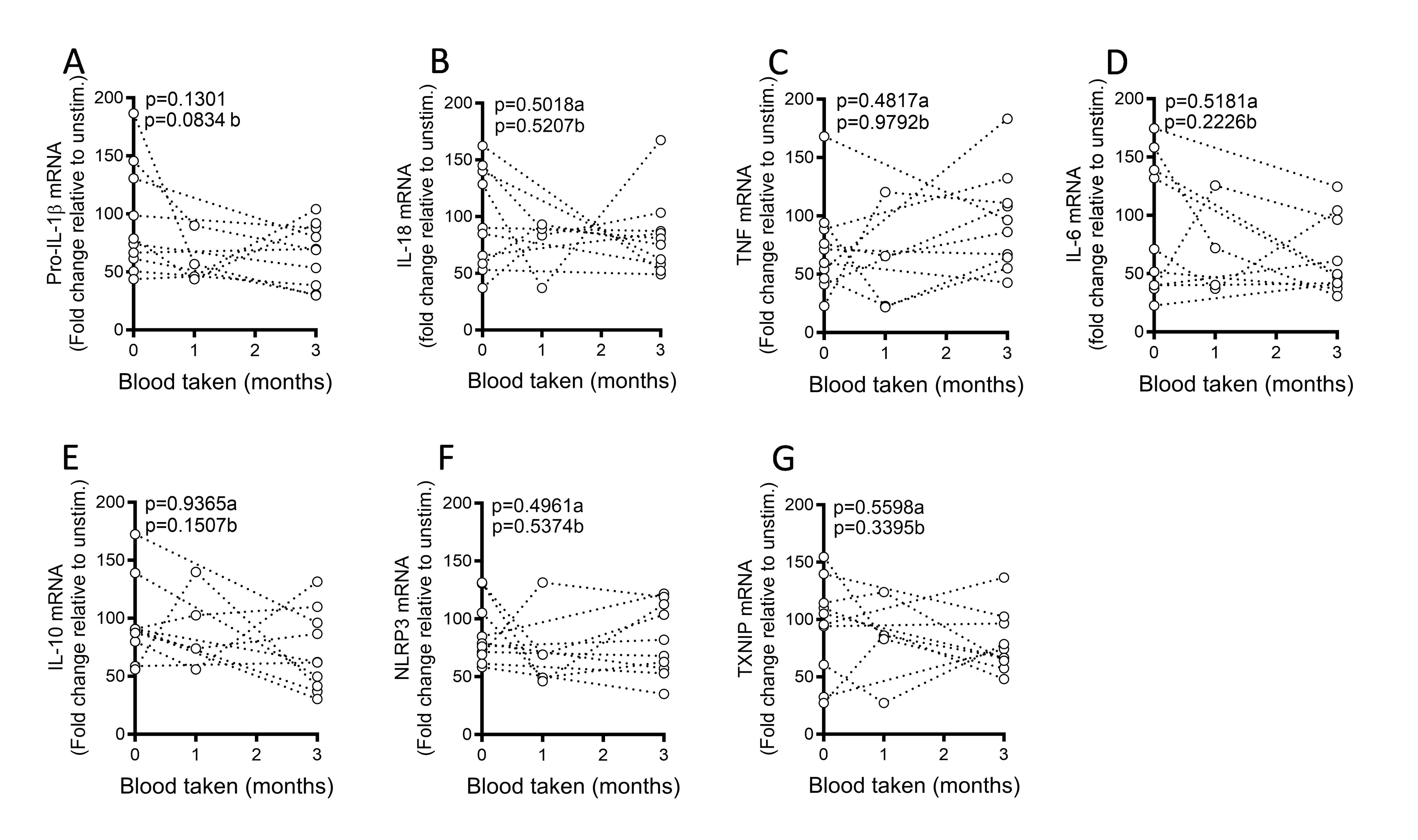

Supplement: S3 Fig — Sera were collected at zero-month, one month and three month from HC volunteers (n = 10). Following isolation, PBMCs were immediately stimulated with LPS (10ng/mL, 4hr), and ATP (5mM) for the final 30 min. RNA was isolated, and mRNA gene expression levels measured A, IL-1β; B, IL-18; C, TNF; D, IL-6; E, IL-10; F, NLRP3; G, TXNIP. A two-way ANOVA statistical test with Tukey’s multiple comparison was performed. P value for baseline to one month (a) and baseline to three months (b) shown on each graph. (TIF) [file pone.0304555.s003.tif]
